# Diagnostic Value and Molecular Function of MicroRNAs in Endometrial Diseases: A Systematic Review

**DOI:** 10.3390/cancers16132416

**Published:** 2024-06-30

**Authors:** Natalia Kluz, Emilia Kowalczyk, Małgorzata Wasilewska, Paulina Gil-Kulik

**Affiliations:** 1 Department of Clinical Genetics, Medical University of Lublin, 11 Radziwillowska Str., 20-080 Lublin, Poland; nataliakluz99@gmail.com; 2 Department of Physical Chemistry, Institute of Chemical Sciences, Maria Curie-Sklodowska University, Maria Curie-Sklodowska Sq. 3, 20-031 Lublin, Poland; malgorzata.wasilewska@mail.umcs.pl

**Keywords:** microRNA, endometrial cancer, recurrent implantation failure, endometriosis

## Abstract

**Simple Summary:**

We have performed a systematic review of the literature about the expression of miRNAs in the endometrium and their potential regulatory roles under pathological conditions such as endometriosis, recurrent implantation failure and endometrial cancer. Our systematic review focused on the potential of miRNAs as non-invasive biomarkers for diagnosing and prognosing endometrial cancer (EC), guiding surgical therapies, and enhancing understanding of EC carcinogenesis.

**Abstract:**

The human endometrium experiences significant cyclic morphological and biochemical changes throughout the menstrual cycle to prepare for embryo implantation. These processes are meticulously regulated by ovarian steroids and various locally expressed genes, encompassing inflammatory reactions, apoptosis, cell proliferation, angiogenesis, differentiation (tissue formation), and tissue remodeling. MicroRNAs (miRNAs) have been recognized as crucial regulators of gene expression, with their altered expression being linked to the onset and progression of various disorders, including cancer. This review examines the expression of miRNAs in the endometrium and their potential regulatory roles under pathological conditions such as endometriosis, recurrent implantation failure and endometrial cancer. Given miRNAs’ critical role in maintaining gene expression stability, understanding the regulatory mechanisms of endometrial miRNAs and identifying their specific target genes could pave the way for developing preventive and therapeutic strategies targeting specific genes associated with these reproductive disorders.

## 1. Introduction

Endometrial cancer (EC) is amongst the cancers with a notable rise in incidence rates. Epidemiological indicators place EC as the sixth most prevalent gynecologic malignancy [1]. The estimated 5-year survival rate stands at approximately 83% [2]. EC risk factors include the following: obesity, diabetes, excess estrogen, hereditary syndromes and exposure to metalloestrogens (Cd, Pb, Cr, and Ni) [3]. 

Despite a frequent early diagnosis and favorable post standard surgical treatment prognosis, EC remains the sole gynecologic cancer experiencing an increasing mortality rate. New molecular prognostic biomarkers for pathogenesis, metastasis or chemo-resistance are essential for better outcome prediction and distinguishing among high and low-risk EC and other endometrial pathologies [4,5].

Endometrial carcinoma typically manifests in postmenopausal women; however a notable 14% of cases present in premenopausal women, and 5% affect patients under the age of 40. Approximately 67% of diagnoses are identified at an early stage, with the cancer localized within the uterus, but 21% exhibit regional dissemination to pelvic lymph nodes or adjacent structures, while around 8% manifest distant metastases [6]. 

EC has been categorized into two main clinicopathological and molecular types: type I (estrogen-dependent) and type II (estrogen-non-dependent). Type I, primarily constituted by endometrioid adenocarcinoma (70–90%), correlates with obesity, excess estrogen, and hormone-receptor positivity [6,7]. Histopathologically, it corresponds with endometrioid endometrial cancer (EEC), characterized by low-grade, low-stage tumors with a favorable prognosis [8]. In contrast, type II, prevalent in older, non-obese women, includes high-grade non-endometrioid subtypes like serous, clear-cell, and undifferentiated carcinomas with an unfavorable prognosis [4,7]. The molecular classification of EC created by the Cancer Genome Atlas Research Network (TCGA) has identified four tumor subtypes: (I) tumors characterized by POLE mutations (ultra-mutated), (II) tumors exhibiting microsatellite instability, (III) tumors with high copy numbers predominantly featuring TP53 mutations, and (IV) the remaining group devoid of these specific alterations [7]. In addition, type I EC is associated with genetic alterations in KRAS, PIK3CA, CTNNB1, PTEN mutations, MLH1 promoter hypermethylation, and microsatellite instability (MSI) [6,9,10]. Serous carcinomas of type II are typically characterized by HER2 amplification and TP53 mutations [4].

Recent advancements in miRNA biology highlight these 18–25 nucleotide-long non-coding RNA molecules as potential targets for innovative methods of diagnosis, prognosis and cancer therapies [4,6,9]. MiRNAs participate in epigenetic and post-transcriptional gene expression regulation, which is crucial in various cellular processes, including apoptosis, proliferation, and cell differentiation. MiRNAs actively engage in various biological processes, prominently contributing to all stages of tumor cell invasiveness and metastasis, encompassing migration, local invasion, epithelial–mesenchymal transition (EMT), and systemic circulation. Notably, some miRNAs can promote tumor growth as oncomiRNAs, while others participate as tumor suppressor miRNAs [1,9]. Moreover, the presence of miRNAs in easily accessible bodily fluids such as serum, plasma, stool, urine, and saliva facilitates straightforward detection [3,9]. 

Endometriosis, which is another very common endometrium disease, is a chronic inflammatory disease underlying the presence of endometrial tissue in an abnormal location (outside the uterus). There are three manifestations of endometriosis, namely, superficial endometriosis, ovarian endometriosis and deep infiltrative endometriosis. It can cause severe pelvic pain and lead to infertility [11]. The mechanism of the pathogenesis of endometriosis is still unclear, but there are theories that speak of retrograde menstrual blood flow and implantation theory, coelomic theory and transformation of healthy tissue into ectopic tissue, pelvic inflammation and immune factors, development of pseudo-cysts and endometriomas, disruption of sex hormone receptors, and epigenetic factors [12,13]. 

Associated with but not limited to endometrial disorders is another disorder—recurrent implantation failure (RIF). It affects about 10% of couples undergoing in vitro fertilization (IVF) and consists of the failure of embryo implantation after the transfer of several good quality embryos [14]. The pathogenesis of the problem may lie in an immunological cause, hematological disorders, endometrial disorders, microbiome disturbances, anatomical or functional abnormalities of the reproductive organs or embryo malformations [15].

The role of miRNA molecules in the pathogenesis of the mentioned endometrial disorders is an ongoing object of research and is still being explored. Studies unveiled numerous relevant miRNAs and their mechanisms potentially involved in endometrial pathogenesis. Researchers described altered miRNA expression profiles in endometrial adenocarcinoma, involving the upregulation of miR-200, miR-205, miR-210, miR-135a, and miR-135b, and the downregulation of miR-133a and miR-133b [16]. Another study stated that highly-expressed miRNA-486-5p stimulates proliferation, migration and invasive activities of EC cells by targeting MARK1 [17]. 

This systematic review aimed to underscore the regulatory role of individual miRNA molecules in endometrial diseases development, summarizing existing knowledge on their mechanisms of action at different tumor development stages. The focus is on their potential as non-invasive biomarkers for diagnosing and prognosing EC and endometriosis, guiding surgical therapies, and mainly enhancing the understanding of EC carcinogenesis.

## 2. Methods

### 2.1. Protocol

Although a formal review protocol was not pre-published for this study, the methodological approach was guided by the Preferred Reporting Items for Systematic Reviews and Meta-Analysis Protocols (PRISMA-P) and the Joanna Briggs Institute’s scoping review guidelines. The reviewer revised and tailored the protocol components to fit the review’s objectives, basing the report items on the PRISMA extension for Scoping Reviews (PRISMA-ScR). This systematic review has been registered: http://osf.io/9pngb (accessed on 28 May 2024).

### 2.2. Data Sources

A thorough electronic search was conducted by the reviewer across multiple databases to find evidence on the subject: PubMed (Medline), Web of Science, and Scopus. The PICO statement focused on population, intervention, comparison, and outcomes. The population was defined as women or patients with endometrial diseases (endometrial cancer, recurrent implantation failure, and endometriosis). The intervention was defined as the microRNA expression status in patients with endometrial diseases. The comparison was defined as the association of clinic-pathological features with microRNA expression. The outcome was defined as the diagnostic value and molecular function in endometrial diseases. The search did not extend to additional sites for unpublished sources or gray literature, focusing instead on peer-reviewed, methodologically robust evidence. The search strategy employed various combinations of MeSH terms and Boolean operators, combining multiple formats and synonyms of the two main concepts: microRNA and endometrial cancer. The search strategy was slightly adjusted to accommodate the features of each database or optimized to yield the broadest range of results while maintaining relevance to the topic. The search covered publications between 2012 and 2023. Specific keywords used included microRNA, miRNA, endometrial cancer, EEC, recurrent implantation failure, and endometriosis. Each concept was represented by multiple synonyms and abbreviations, using Boolean operators OR and AND to ensure the comprehensive retrieval of relevant sources from the targeted databases. The search strategy was slightly adjusted to fit the specific features of each database and to maximize the relevant results. For example, the search string used in the PubMed database was as follows: 

(microrna OR mirna) AND (endometrial cancer OR EEC OR recurrent implantation failure OR endometriosis).

### 2.3. Eligibility Criteria

As a scoping review, this study employed inclusive eligibility criteria to identify the maximum number of relevant sources on the role of microRNAs in endometrial diseases, especially endometrial cancer. The analysis covered the detection, characterization, quantification, and functional analysis of microRNA components in endometrial disorders, facilitating the identification of microRNA types, quantities, and functions. Papers published in languages other than English were excluded to ensure review accuracy and avoid potential translation errors. Additionally, narrative review articles, book chapters, and conference papers were excluded to ensure the inclusion of only high-level primary evidence sources.

### 2.4. Source Selection

**Figure 1:**
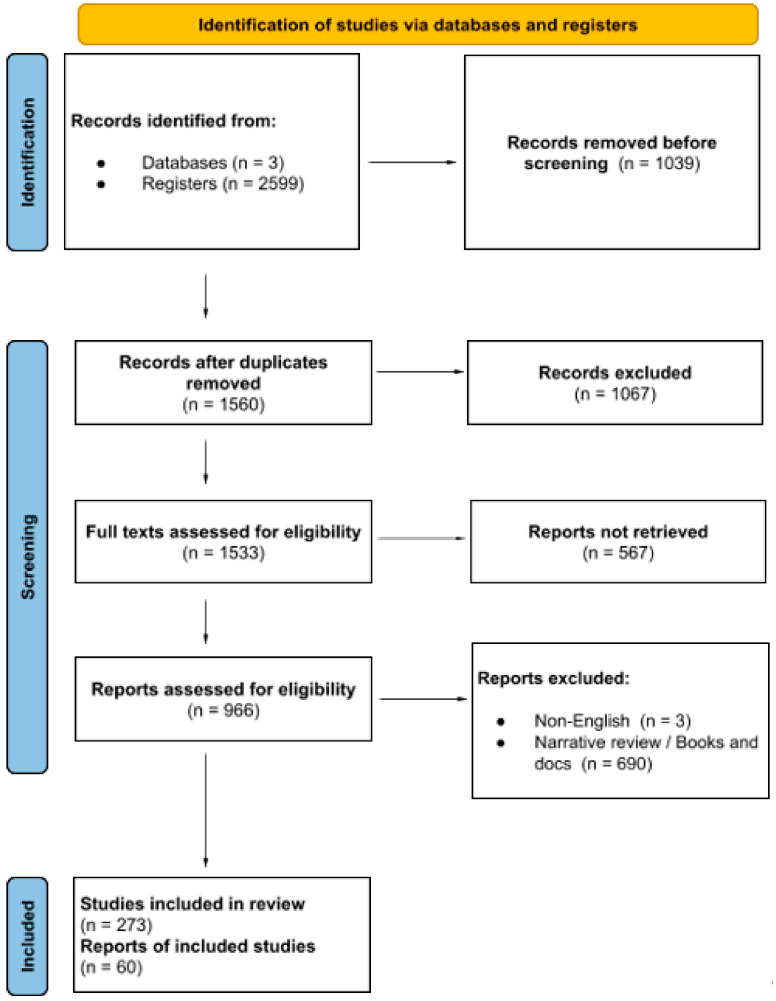
PRISMA flow diagram [18].

The reviewers screened the retrieved sources in two phases. In the first phase, duplicates were removed using free online software and then manually double-checked. After duplicate removal, the titles, abstracts, and keywords of the sources were screened to identify those mentioning the two main concepts and to exclude non-English sources. In the second phase, the abstracts of the remaining sources were reviewed to determine their relevance to the review topic and to identify whether microRNA analysis was performed. This screening helped the reviewers select articles with methodologies aligned with the review’s aims and inclusion criteria. Articles with unclear methodologies were further examined by the reviewer using Section 2. Finally, the reference sections of the selected articles were reviewed to identify additional potential sources for inclusion (see Figure 1). 

### 2.5. Quality Assessment

Two reviewers rigorously examined the chosen articles to determine whether they met the predetermined inclusion criteria. Each article underwent detailed quality and source database analysis. The selected articles were indexed in PubMed. The Mixed Methods Assessment Tool (MMAT) [19] was used to assess the quality of each study for this systematic literature review, ensuring a comprehensive evaluation of both the qualitative and quantitative studies. The quality of the various included studies as assessed by the MMAT is shown in Table 1.

## 3. Results

The initial database search yielded 2599 results, which were filtered in the first phase by removing duplicates and unrelated topics. In the next phase, 966 sources underwent full-text analysis for relevance and methodological rigor, leading to the exclusion of 693 sources. Screening the reference sections identified two additional sources, resulting in a total of 60 articles included in the scoping review (Figure 1).

**Figure 2:**
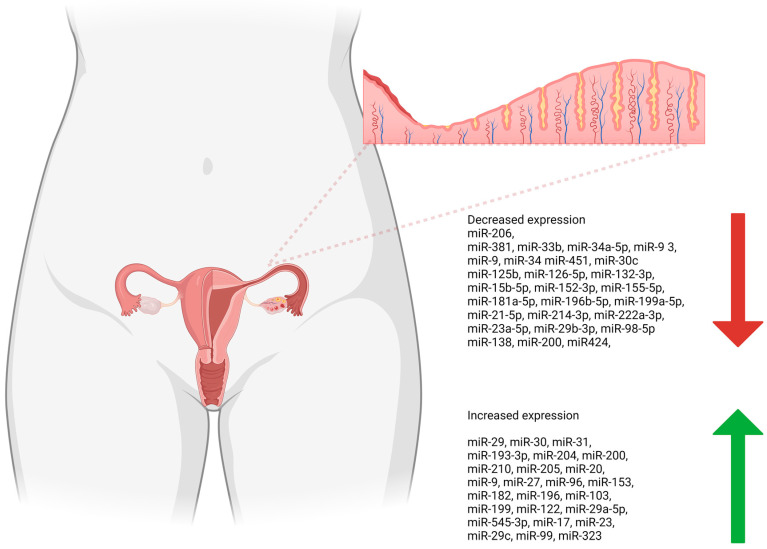
A summary of miRNAs reported in recent studies considering their role in the pathogenesis of the most noticeable endometrial diseases. (Created in BioRender.com (accessed on 28 May 2024), license number GH26XHCAKS).

The majority of the studies only included endometrial cancer, 20 were about endometriosis and 8 articles mentioned recurrent implantation failure and ectopic pregnancy. As shown in Table 1, the studies on the subject can be categorized into trending and function of microRNAs based on the research focus. There were 25 different miRNAs identified as significantly upregulated and 27 miRNAs identified as downregulated (Figure 2, Table 2).

### Risk of Bias

We used the Newcastle–Ottawa Scale (NOS) [53] to assess the quality of the included case–control and cohort studies. For this purpose, the authors evaluated the eligible articles: Selection (four elements), Comparability (one element), and Results (three elements). Each study could receive a maximum of one star for each numbered item in the Selection and Exposure categories. A maximum of two stars could be awarded for comparability. The score consisted of eight elements, including, depending on the type of study: appropriate case definition, representativeness of cases, control selection, control definition, comparability, exposure determination, same method and non-response rate or representativeness of the exposed cohort, selection of the non-exposed cohort, ascertainment of exposure, demonstration that the outcome of interest was not present at the start of the study, comparability of the cohorts on the basis of the design or analysis, assessment of outcome, was follow-up long enough for outcomes to occur, and adequacy of follow-up of cohorts. The total quality score ranged from zero to nine. Studies assessed with a score of ≥5 were considered to be at low risk of bias. The Newcastle–Ottawa scoring results are shown in Table 3. All of these studies were rated as having a low risk of bias (Table 3).

Newcastle–Ottawa Quality Assessment Scale: Case–Control Studies: 1. adequate case definition; 2. representativeness of cases; 3. selection of controls; 4. definition of controls; 5. comparability; 6. ascertainment of exposure; 7. same method; 8. non-response rate.

Newcastle–Ottawa Quality Assessment Scale: Cohort Studies: 1. representativeness of the exposed cohort; 2. selection of the non-exposed cohort; 3. ascertainment of exposure; 4. demonstration that outcome of interest was not present at start of study; 5. comparability of cohorts on the basis of the design or analysis; 6. assessment of outcome; 7. was follow-up long enough for outcomes to occur; 8. adequacy of follow-up of cohorts.

## 4. Discussion

### 4.1. Regulatory Functions

MiRNAs are a class of non-coding RNA molecules that regulate gene expression after transcription, usually by incomplete complementary binding to the 3′-non-translational region (3′-UTR) of the target transcript, resulting in degradation of the mRNA molecule or inhibition of translation [54,55]. The miRNA-mediated gene expression is regulated by cytoplasmic granules containing ribonucleoproteins (RNPs), which consist of factors related to the degradation of mRNA and miRNA. In most cases, the transcript is inhibited by complementarity between miRNA and mRNA, resulting in cleavage or muting of the translational mRNA. Mature miRNAs can also reduce gene expression by coupling the bases of mature miRNA to their target mRNA, a regulatory mechanism affected by the incidence of the target mRNA [56]. 

To date, more than 2,500 miRNAs (miRBase v.20) have been discovered in human cells, and the number continues to rise. It is estimated that these molecules regulate up to 60% of the genes encoding proteins, making miRNA one of the most important genetic modulators in mammals [57]. The importance of microRNA in tumors is related to their regulation of these cellular processes and numerous signaling pathways, including the cell cycle, proliferation, programmed cell death, cellular stress response, p53 signaling pathway, and mTOR signaling pathway [58]. The same miRNAs may play opposite roles in different tumors by promoting tumor growth (oncomiRNA) or acting as tumor miRNA suppressors. By targeting the 3′-UTR region (non-translatable) of several mRNAs, they regulate all cancer characteristics defined by Hanahan and Weinberg: proliferation, angiogenesis, and invasion, and also influence the therapeutic resistance of cancer cells [1]. 

A deviation of miRNA expression in pathological situations may be both a cause and a result of an existing pathology. The complexity of miRNA activities and the variety of signaling pathways controlled by many miRNAs requires a thorough and rigorous analysis, so this review focuses on some of the most important points. MiRNAs, which are involved in the regulation of processes such as inflammation, hypoxia, angiogenesis and cell proliferation that are common in the pathophysiology of endometriosis and endometrioid endometrial carcinoma, can be an important diagnostic tool, as well as the possible links between these diseases and other genetic and epigenetic factors. Up to 754 neoplastic miRNAs have been identified in the endometrium so far, some of which correlate with histological type, advanced FIGO stage, lymph node involvement or metastasis [59]. 

There is growing evidence that miRNAs play an important role in the development of the endometrium during the menstrual cycle in humans. To begin with, the dependence of miRNA regulation in the endometrium on sex hormones should be highlighted. Conversely, miRNAs have been shown to regulate the action of these hormones by influencing cell receptors. The profiling of miRNA in the endometrium varies according to the stage of the menstrual cycle, depending on the change in the status of these steroidal sex hormones, as shown by Kuokkanen et al. In their study, significant differences in miRNA expression in human endometrial cells were noted from late proliferation and the luteal phase. Twelve miRNAs (miR-29B, miR-29C, miR-30B, miR-30D, miR-31, miR-193a-3p, miR-203, miR-204, miR-200C, miR-210, miR-582-5P, and miR-345) were found to be significantly upregulated in luteal phase samples, suggesting that miRNAs play an important role in reducing the expression of cell cycle genes in the mid-secretory phase of the endometrium, thus inhibiting cell proliferation [20]. 

ERα, the estrogen receptor, is an important protein for predicting the efficacy of therapy in patients with type 1 EC. Amplification of the gene encoding this receptor is considered a common occurrence in endometrial cancer, so inhibition of its expression by miRNA-206 was considered an antiproliferative and anti-invasion effect [21]. 

Epigenetics play a crucial role in endometrial cancer. Mute-switching of repair genes and DNA by DNA hypermethylation has been observed [37,60]. One example is miRNA-205, which is involved in the regulation of PTEN expression, the most frequently modified gene in EC. PTEN has an important inhibitory function by promoting apoptosis and proliferation, and its deletion or mutation leads to carcinogenesis. mRNA PTEN is negatively regulated by miR-205 [22,23]. PTEN is a suppressor gene that negatively regulates the PI3K-AKT signaling pathway. This pathway is responsible for the most important functions such as survival, growth, proliferation and apoptosis [61]. Mutations responsible for the dysregulation of gene expression, which encode the proteins of this pathway, also lead to oncogenesis in EC. 

The transcription factor FOXO1 is another target of the PI3K pathway and can modulate the different biological processes involved in the endometrial phases, such as uterine cell regeneration and differentiation, tissue remodeling and menstruation [62]. It is proven that modulation by the described miRNAs also takes place at the FOXO1 mRNAs level—in EC type 1, four mutated molecules are responsible for the downward regulation, i.e., miR-9, miR-27, miR-96, miR-153 and miR-182 [24]. As a result, apoptosis is suppressed and the tumor process is promoted. 

Recent studies have also shown a different correlation associated with the described interaction between miRNA and PI3K. The IGF1R epithelial receptor, which binds its IGF1 and IGF2 ligands, is able to activate the PI3K pathway by stimulating cell proliferation and inhibiting apoptosis [63]. IGF1R has been shown to be strongly expressed in endometrial carcinoma tissue and is inversely correlated with miR-381 levels [25]. Therefore, when IGF1R is overexpressed, as with most tumors, it plays an anti-apoptotic role by promoting cancer cell survival and tumor metastases. 

MiRNAs may also be responsible for the metastasis potential of endometrial cancer. E-cadherin is a calcium-dependent transmembrane adhesion molecule of the epithelium responsible for cell coherence. E-cadherin is also crucial for the oncogenesis of the epithelial–mesenchymal transition (EMT) [64]. Decreased expression of this protein by miRNA-205 was associated with reduced intercellular adhesion and thus increased metastatic potential and deep myometrial invasion in EC. The process of EMT activation, i.e., the transformation of endometrial epithelial cells into migrating mesenchymal cells and other miRNAs, is also responsible. EMT is regulated by the mesenchymal transcription regulator FOXF2. It has been confirmed that this factor is modulated by miRNA-182. Mutated miRNA may promote tumor growth by reducing FOXF2 expression [26]. These small transcripts are also involved in regulating cell cycle control by modulating checkpoints and DNA repair mechanisms. Data from studies by Yao et al. showed that miRNA-182 promoted the proliferation of tumor cells in EC and that downregulation of miRNA resulted in a stop of the cell cycle in the G1 phase, reducing the ability to form colonies and inhibiting tumor growth [27].

### 4.2. Inflammatory Response

During the menstrual cycle, the endometrium tissue undergoes changes that are controlled by released sex hormones and numerous genes. This leads to apoptosis, cell proliferation, angiogenesis and tissue transformation. Processes in the endometrium resemble an inflammatory reaction in which humoral factors such as growth factors, chemokines, inflammatory cytokines, proteases and extracellular matrix metalloproteinases (MMPs) are produced and increased [65]. The changes during the monthly cycle are associated with the expression of substances that are highly inflammatory and induce immunological mechanisms. Research shows that small non-coding RNAs serve as regulatory molecules in these processes [35]. Several miRNA molecules have been found to significantly influence the gene expression of pro-inflammatory molecules and the development of immune cells, i.e., T and B lymphocytes and dendritic cells [33,35]. The inflammatory response of the endometrium is influenced by miRNA molecules such as let7, miR-17-5p, miR-20a, miR-106a, miR-125b, miR-146 and miR-155. It is worth noting that the expression of miR-20a, miR-21, miR-23, miR-26a, miR-18a, miR-181a, miR-206 and miR-142-5p is significantly regulated by the sex hormones: estradiol-17B and progesterone [65,66]. 

In one study examining the expression profiles of microRNAs (miRNAs) linked to inflammation in endometrial stromal cells (ESCs) from patients with and without endometriosis, it was found that several miRNAs were significantly downregulated in patients with endometriosis. Specifically, miR-126-5p, miR-132-3p, miR-15b-5p, miR-152-3p, miR-155-5p, miR-181a-5p, miR-196b-5p, miR-199a-5p, miR-21-5p, miR-214-3p, miR-222a-3p, miR-23a-5p, miR-29b-3p, and miR-98-5p were all significantly reduced in the ESCs of endometriosis patients compared to those without the condition [34,35].

The mentioned types of miRNAs and in particular miRNA-125b and miRNA-155 are involved in the regulation of the expression of transforming growth factor β (TGF-β), the TGF-β receptor, the estrogen and progesterone receptors, and CYP-19A1 aromatase [33]. miRNA-155 is additionally responsible for the expression of proteins involved in the lipopolysaccharide (LPS) signaling pathway and the upregulation of tumor necrosis factor α (TNF-α). Conversely, miR-125b suppresses the expression of TNF-α. The opposing actions of miR-125b and miR-155 under normal conditions are therefore fundamental to the regulation of the inflammatory state and immune response in the endometrium. Under normal physiological conditions, microRNAs (miRNAs) create a negative feedback mechanism to mitigate excessive inflammatory responses within the nuclear factor kappa-light-chain-enhancer of activated B cells (NF-kB) pathway. However, the altered expression of miRNAs targeting this pathway disrupts the regulation of inflammation, potentially contributing to carcinogenesis [33,65]. 

In a prospective study by Mu F. et al., elevated levels of IL-1β were shown to be associated with an increased risk of endometriosis. Additionally, IL-6, which is strongly expressed by macrophages, influences cell migration, a key factor in the development of endometriosis [28,67]. The downregulation of miR-138 expression enhances inflammation in endometriosis by elevating levels of TNF-α, IL-1β, IL-6, and IL-18 through the activation of the NF-κB signaling pathway and VEGF. Additionally, the upregulation of miRNA 125b-5p and the downregulation of let-7b-5p have been shown to correlate with elevated levels of proinflammatory cytokines in women with endometriosis [34,35].

The overexpression of miR-20a in endometriotic lesion cells has also been observed to impact the production of inflammatory factors. miR-20a contributes to oxidative stress and increased production of prostaglandin PGE2 by reducing the production of the dual-specificity phosphatase (DUSP-2) and enhancing the activity of extracellular signal-regulated kinases (ERK) [36].

The role of miRNA dysregulation in the epigenesis of endometrial carcinoma is particularly important in dysfunctional uterine hemorrhage, during which significantly increased inflammatory activity is observed in relation to the normal menstrual cycle. There is an increased expression of pro-inflammatory mediators (especially TNF-α) and an increase in NF-kB activity. However, dysregulated expression of miRNA results in hyperactivation of inflammatory genes during the inflammatory response. In addition, increased NF-kB activity by inflammatory monocyte stimulation leads to the induction of miRNA-146, which is shown to be a suppressor factor for the expression of tumor necrosis factor receptor associated factor 6 (TRAF6) and interleukin-1 receptor-associated kinase 1 (IRAK1). The described relationship represents a negative feedback loop that modulates inflammation in the endometrium. TRAF6 and IRAK1 regulate the toll-like receptor (TLR) downward and suppress the production of anti-inflammatory cytokines [34,66].

During bacterial or viral infections as well as during the menstrual cycle (especially in dysfunctional bleeding), there is also an increase in the expression of matrix metalloproteinases (MMPs) [33]. In order to prevent the degradation of collagen, fibrinogen and fibronectin by MMPs, the activity of metalloproteinase inhibitors (TIMP) in the tissue is important. miR-200b has been shown to target TIMP-2 expression, miR-200c targets FN1 (the fibronectin-coding gene) expression, and both miRNAs act as suppressive miRNAs in cancer cells. On the other hand, miR-103 targets the expression of TIMP-3 and stimulates tumor growth and invasion. It turns out that the regulation of extracellular matrix remodeling (ECM), which takes place during the inflammatory process, is particularly important for the invasion of tissue by the developing tumor [1].

In the normal menstrual cycle, the next step is the repair phase. For it to occur properly, the inflammation must be silenced by reducing the number of cells and inflammatory mediators. Dysregulation of miRNA and excessive expression of pro-inflammatory cytokines, which amplifies the inflammatory response, impair this process and impair the regeneration of endometrial tissue. Such a condition may promote the beginning of the process of carcinogenesis [33,34]. As the collected data show, the expression of one gene can be regulated by several miRNAs, and also miRNAs may play a role in regulating invasion by different mRNAs [1]. This was evident in a recent prospective study, which demonstrated that both miR-199a and miR-122 were upregulated in the serum of one group of women with endometriosis and, conversely, a significant downregulation of miR-199a was observed in another group [28,36].

### 4.3. Angiogenesis

The process of angiogenesis plays an important role in the formation and growth of endometrial tumors. In the normal cycle it takes place in the corpus luteum, during placental development and in the cyclical remodeling of the endometrium. However, this process should be self-limited due to the proper production of angiogenic factors and their inhibitors by the endometrium [33,37]. Dysregulated expression of miRNAs, observed in endometrial cancer, has a significant influence on the angiogenesis process. miRNAs have a bi-directional effect on angiogenesis. On the one hand, miRNAs may target the expression of genes involved in angiogenesis, and on the other hand, angiogenic factors, i.e., vascular endothelial growth factor (VEGF-A) or anti-angiogenic factors, i.e., thrombospondin-1 (TSP-1), may influence the expression of miRNAs [1,28]. 

Recent studies have identified miRNAs—miR-29a-5p and miR-545-3p—that also target the expression of VEGF-A and influence the progression of endometrial cancer by stimulating angiogenesis [1]. Additionally, it has been demonstrated that endometrial mesenchymal stem cells treated with overexpressed miR-199a-5p also exhibited reduced VEGFA expression, as well as decreased cell proliferation and angiogenesis [28,37]. 

In contrast, the downregulation of miR-424 expression is observed in endometrial cancer cells. This molecule influences the activity of VEGFA, ERBB, mTOR, TGF-β, and the PTEN/PI3K/AKT pathway, thereby affecting the process of tumor angiogenesis [38]. 

Similarly, the molecule miR-15a-5p has been found to be involved in the downregulation of VEGF-A, which was significantly lower in the cells of patients with endometriosis compared to the control group [41,42]. The negative regulation of angiogenesis and tumor growth in endometrial cancer (EC) by downregulating vascular endothelial growth factor A (VEGFA) was also observed with miR-29b-3p. This miRNA mediates its effects by inactivating the MAPK/ERK and PI3K/AKT pathways [32,39]. 

It is also known that numerous miRNAs are involved in EC invasiveness and metastasis via, for example, the epithelial–mesenchymal transition (EMT) or systemic circulation [1,34]. Both EMT and angiogenesis play crucial roles in the pathogenesis of endometrial lesions. Nothnick et al. demonstrated a link between dysregulated miRNAs and the processes of EMT and angiogenesis, involving the following molecules: miR-15, -20a, -23a/b, -29c, -126, -142, -145, -183, -199a, and -451 [28,68]. It has also been proven that the upregulation of miR-21-5p promotes EMT. In contrast, the downregulation of miR-21-5p suppresses EMT in endometrial cancer cell lines [43]. In addition, miR-29b, miR-148b, miR-194 and miR-214-3p were identified to inhibit the development of tumor metastasis, while miR-652 promotes this process [1]. It also appears that upregulation of miR-576-5p promotes proliferation and enhances EC cell metastasis by inhibiting the expression of zinc finger and BTB domain containing four (ZBTB4) [5]. 

The specific mechanism of action of miRNA-20a in inducing angiogenesis is the inhibition of the expression of DUSP2 and the induction of hyperexpression of genes related to ERK. ERK is a kinase of the group of mitogen-activated kinases (MAPs) that are involved in the activities of most non-nuclear oncogenes. ERK acts on numerous growth factors, including VEGF-A, whose hyperexpression leads to increased proliferation and angiogenesis in cancer cells in the endometrium. miRNA-20a can therefore trigger cancer development by expression of the described genes [33].

A review of the research significantly suggests that many miRNAs may be responsible for dysregulated angiogenesis and the development of endometrial cancer. Their role in the pathogenesis of cancer is supported primarily by the correlation between miRNA and VEGF-A, but as can be seen, small non-coding RNAs also influence the expression of many other angiogenic and antiangiogenic factors.

### 4.4. Disorders

#### 4.4.1. miRNAs in Endometriosis

Recently, new modulators of gene expression—microRNAs (miRNAs)—have been discovered, and their stability and specificity might lend significant potential biomarker value for endometriosis [35,69,70]. Epigenetic dysregulations, particularly miRNAs, are thought to contribute to the development of endometriosis. These small RNA molecules interact with target mRNAs and influence processes such as hypoxic injury, survival, proliferation, inflammation, remodeling, angiogenesis, and steroidogenesis, including progesterone resistance. Consequently, miRNAs have the potential to be non-invasive biomarkers and targeted therapies for endometriosis [35,71]. Hormonal and immunological factors should be comprehensively investigated. The complexity of the intracellular interactions between the signaling pathways suggests that miRNAs may be involved in the pathophysiological mechanisms of endometriosis, as they are involved in the regulation of numerous cellular processes.

In 2009, Ohlsson Teague et al. conducted the first study that found a link between miRNA and endometriosis. In ectopic and eutopic endometrial tissue, 14 upregulated miRNAs and 8 downregulated miRNAs were identified [44]. VEGF A stimulates angiogenesis and is involved in the formation of blood vessels in endometriosis, so miRNAs may indirectly be a predictor of the progression of the described disease. In their meta-analysis, Bjorkman et al. referred to the most commonly identified target for endometriosis-associated miRNAs, vascular endothelial growth factor A (VEGF A), regulated by five miRNAs (miR-15a-5p, miR-33b, miR-34a-5p, miR-9 3, and miR-199a) [28]. In another study, the authors showed that miR-9 and miR-34, which are thought to play a key role in inhibiting p53-suppressor protein-dependent proliferation, were decreased in endometriosis patients. One of the regulatory targets of miR-9 is FOXP1, a gene that contains information about an anti-apoptotic protein known to be overexpressed in the endometrium of patients with the disease described in this chapter [29]. Another, miR-451, was also found in the endometrium in women with endometriosis compared to healthy patients. As a result, apoptosis is inhibited and the proliferation of endometrial cells is promoted [30]. Therefore, it has been suggested that deregulation of these miRNAs and their target genes involved in anti-apoptotic pathways may contribute to a proliferative phenotype in the luteal phase of the menstrual cycle in women with ectopic endometrium. 

Consistent with these findings, analysis of the ectopic endometrium in women with endometriosis showed that miR-196a is also overexpressed compared to the control group, i.e., healthy women. MiR-196a regulates the MEK/ERK signaling pathway in the cell, which triggers the immune response and inflammatory responses [31]. PAI-1 plasminogen-1 activator inhibitor, also known as endothelial plasminogen activator inhibitor, is the main source of intravascular fibrinolysis. Furthermore, it is related to the regulation of cell adhesion and migration, which plays a crucial role in the pathogenesis of endometriosis [72]. In Chen et al.’s study, miR-30c levels were shown to be reduced compared to healthy endometrium. This downward regulation was associated with increased expression of this plasminogen activator inhibitor type 1 (PAI-1), which was associated with increased proliferation, migration, and invasion capability of dysfunctional endometrial cells [40]. Experiments to profile miRNA expression showed an additional hormonal relationship that plays a key role in the pathogen of the described disease. The increased expression of miR-29c in endometriotic tissue was associated with progesterone resistance in the cells [44]. Moreover, in another study, the increased expression of miR-143-3p enhanced the motility of endometriotic lesions, promoting disease progression due to an inadequate response to progesterone treatment [46]. The target of miRNA-29c is a progesterone-regulated protein FK506 (FKBP4), which is responsible for changes in the functional layer of the uterine mucosa that reacts to the secretory activity of the corpus luteum by the implanting blastocyst. This differentiated regulation suggests that miRNAs may serve as channels for impaired progesterone activity in endometriosis [45].

It is crucial to point out that microRNAs also play pivotal roles in adenomyosis—a separate study indicated that expression of miRNA-17 was increased in the endometrial tissues of patients with adenomyosis and might influence cell apoptosis and cyclin expression through the targeted downregulation of the gene phosphatase and tensin homolog (PTEN). These findings suggest that miRNA-17 may promote the occurrence and/or development of adenomyosis [47].

In summary, processes such as proliferation, inhibition of apoptosis, angiogenesis, extracellular matrix transformation, cellular invasion, inflammation signaling and hormone regulation are documented by studies on miRNA molecules involved in endometriosis.

#### 4.4.2. miRNAs in Recurrent Implantation Failure (RIF)

Recurrent implantation failure (RIF) means that after implantation failure >3 failed embryo transmissions, pregnancy is not present and up to 50% of couples undergoing in vitro fertilization (IVF) or intracytoplasmic sperm injection (ICSI) are observed to be affected by RIF [73].

Identified causes of RIF include abnormal uterine anatomy, genetic abnormalities, sperm-related dysfunction, thrombophilia, hormonal and metabolic pathologies, autoimmune diseases and overactivation of the uterine immune profile [74]. It is generally accepted that miRNAs are involved in adhesive interactions on the cell surface and act as mRNA regulators of genes involved in endometrial transformation. As a result, they are involved in the implantation of the blastocyst. Some studies compared the miRNA expression profiles of infertile endometrium (study group) and fertile women (control group) in the luteal phase. Thirteen miRNAs differed, including a double upregulation of miR-23b, miR-99a, and miR-145. The expression of the last miR is oriented to the mRNA, which contains the IGF1R information. In addition, the mRNA expression of the adhesive molecules, i.e., N-cadherin, netrin-4, H2AFX, as well as the cell cycle and Wnt-signal molecules critical for embryogenesis, was found to be low in RIF endometrial samples [74]. Another study compared the level of endometrial miRNA expression during the implantation phase of RIF patients with samples from pregnant and recently delivered patients after a successful embryo transfer trial. Expression levels of up to 105 miRNA were found to vary significantly in the RIF group. The miR-145 described above was a common miRNA that was elevated in endometrial samples from RIF patients [49,50]. The results about different levels of miRNA expression in the endometrium can help clinicians predict the probability of successful implantation and even obtain a risk assessment for RIF before IVF and ICSI. This will increase the chance of choosing the best embryo transfer treatment strategy in the light of RIF and allow for achieving satisfactory clinical success.

#### 4.4.3. miRNAs in Ectopic Pregnancy (EP)

Ectopic pregnancy is one of the most serious complications of endometriosis. EP is a condition in which a blastocyst is implanted outside the uterine cavity. EP remains a serious reproductive health problem, as it carries the risk of mothers dying in the first trimester [75]. Measurement of progesterone and human chorionic gonadotropin (hCG) in serum as well as transvaginal sonography, which serve as diagnostic tools for fallopian pregnancies, are widely used. However, the sensitivity and diagnostic specificity, i.e., the frequency of false-positive and false-negative results for these instruments, is a cause for concern. The hope lies in new molecular biomarkers to help diagnose the disease. 

Pregnancy-related miRNAs have been used to identify complications associated with EP. In one study, upregulation of miR-323-3p expression was observed in PE cases compared to vital intrauterine pregnancies. A combination of the conventional biomarkers hCG, progesterone and miR-323-3p has been reported to significantly improve the diagnostic sensitivity (96.3%) and diagnostic specificity (72.6%). These observations suggest that miR-323-3p may be a candidate EP diagnostic marker [51]. This was also confirmed by another study in which miRNA concentrations (miR-323-3p, miR-515-3p, miR-517a, miR-517c and miR-518b) and serum hCG concentrations differed statistically significantly between women with EP and a single intrauterine pregnancy [52].

However, further prospective and extensive studies are needed to confirm the usefulness of miR-323-3p as a biomarker for EP diagnosis.

### 4.5. Study Strengths and Limitations

Given the challenges of the time-consuming and costly nature of miRNA analysis, our findings highlight the need to prioritize certain miRNAs for predicting and routinely screening EC and other endometrial pathologies. The consistent upregulation of miRNAs such as miR-205 and miR-29 across various studies and populations makes them particularly promising biomarkers for assessing the risk of EC. These miRNAs have shown significant changes in expression levels in endometrial diseases and have been validated through different research methodologies, indicating their robustness and potential clinical utility.

Additionally, the discovery of miRNAs that are significantly downregulated in EC, such as miR-199a-5p and miR-15a-5p, adds another layer to the miRNA profile that could be crucial for predicting carcinogenesis. Given the important role of miRNAs in regulating key physiological pathways in endometrial tissue, such as TGF-beta and p53 signaling, it is recommended that miR-9 and miR-424 be considered primary candidates for further development into routine screening tests for endometrial pathologies, especially EC. Their consistent upregulation in EC cases and association with critical signaling pathways make them viable biomarkers for early detection and intervention strategies.

Implementing screening based on these specific miRNAs could improve our predictive capabilities and enable a more cost-effective allocation of resources. Future research should aim to validate these miRNAs in larger, more diverse populations and develop streamlined protocols for their detection and analysis in clinical settings.

The present systematic review has several limitations. First, we performed an overall systematic review by combining different mRNAs to qualify the association between miRNAs and pathologies of the endometrium, thereby preventing the accurate identification of the specific miRNA that best detects diseases in the selected population. However, this systematic review aimed to offer a comprehensive quantitative overview of the existing evidence in this field rather than a precise directive for healthcare professionals in daily clinical practice. Furthermore, we attempted to pool the evidence to analyze at least the most relevant miRNAs in the studies. Second, the present study’s methodology provides a descriptive synthesis of published associations between specific miRNAs and EC and other endometrial pathologies. This approach did not involve amalgamating the raw data from the primary studies. The high variability in terms of the characteristics of the patients, type of disease, their disease stages, molecular cancer subtypes, and the type of sample used could call into question the plausibility of the results. However, this systematic review aimed to give a quantitative overview of the association between specific miRNAs and endometrial diseases rather than corroborate a validated model for a specific miRNAs’ predictive ability. 

## 5. Conclusions and Future Directions

Recent advancements in molecular biology have greatly improved our understanding of the uterine molecular environment, particularly through identifying gene expression in the endometrium under both normal and diseased conditions. These gene products, functioning independently or through interactive autocrine and paracrine mechanisms, regulate crucial processes such as cell cycle progression, inflammatory and immune responses, differentiation, tissue remodeling, and apoptosis. The precise regulation of gene expression is essential for normal development and the onset and progression of uterine disorders. Dysregulated gene expression is associated with various pathological outcomes, including endometriosis, recurrent implantation failure, infertility, and endometrial cancer. 

MicroRNAs (miRNAs) have emerged as critical regulators of gene expression. Techniques such as cloning strategies, expression profiling, and next-generation sequencing have identified numerous miRNAs, with functional analyses highlighting their significant role in post-transcriptional regulation. Experimental models have provided valuable insights into the biological relevance of miRNAs in various cells and tissues under both normal and diseased conditions. These small, non-coding RNAs regulate gene expression through complementary interactions with their target genes, primarily by transcriptional and translational repression. However, current evidence regarding the expression, regulation, and function of miRNAs in human reproductive tract tissues is limited to a few studies. The expression profile of several hundred miRNAs in the endometrium and endometrial cells, and their aberrant expression in ectopic endometrium in women with endometriosis, underscores their key biological relevance. 

Our systematic review focused on the potential of miRNAs as non-invasive biomarkers for diagnosing and prognosing endometrial cancer (EC), guiding surgical therapies, and enhancing understanding of EC carcinogenesis. Further research is needed to expand our understanding of miRNA functions and their regulatory mechanisms in the reproductive tract. Such studies could provide new insights into the molecular basis of uterine disorders and potentially lead to the development of novel diagnostic and therapeutic strategies.

## Figures and Tables

**Table 1:** Quality assessment of the study by the Mixed Methods Assessment Tool.

Category of Study Designs	Methodological Quality Criteria	Responses			
		Yes	No	Cannot Tell	
Screening questions (for all types)	S1. Are there clear research questions?	✔			
	S2. Do the collected data allow me to address the research questions?	✔			
	Further appraisal may not be feasible or appropriate when the answer is ‘No’ or ‘Cannot tell’ to one or both screening questions.				
Qualitative	1. Is the qualitative approach appropriate to answer the research question?	✔			1–12,14–17,20–22,29,37,40–45, 47–51,53–58,63,65,68,70,73
	2. Are the qualitative data collection methods adequate to address the research question?	✔			
	3. Are the findings adequately derived from the data?	✔			
	4. Is the interpretation of results sufficiently substantiated by data?	✔			
	5. Is there coherence between qualitative data sources, collection, analysis and interpretation?	✔			
Quantitative	1. Is the sampling strategy relevant to address the research question?	✔			13,23–28,30–36,38,39,46,52,59–62, 64,66,67,71,72, 74
	2. Is the sample representative of the target population?	✔			
	3. Are the measurements appropriate?	✔			
	4. Is the risk of nonresponse bias low?	✔			
	5. Is the statistical analysis appropriate to answer the research question?	✔			

**Table 2:** Comparison of the variation of expression of miRNAs in unhealthy patients compared to healthy patients in our systematic review.

Name of miRNA	Trend	Function of microRNA in Healthy Cells	References
miR-29b, miR-29c, miR-30b, miR-30d, miR-31, miR-193a-3p, miR-203, miR-204, miR-200c, miR-210, miR-582-5p, miR-345	Up	Reducing the expression of cell cycle genes in mid-secretory phase of the endometrium	[20]
miR-206	Down	Inhibiting ERα-dependent proliferation of the endometrium, impairing invasiveness and inducing cell cycle arrest of ERα-positive EEC cell lines	[21]
miR-205	Up	Inhibiting PTEN gene expression	[22,23]
miR-9, miR-27, miR-96, miR-153 and miR-182	Up	Downregulation of tumor suppressor FOXO1—uncontrolled proliferation of EC	[24]
miR-381	Down	Downregulation of IGF1R, which plays an anti-apoptotic role by promoting cancer cell survival and tumor metastases	[25]
miR-182-5p	Up	Upregulating FOXF2, RECK and MTSS1 genes—promoting cell invasion and proliferation Activating epithelial–mesenchymal transition	[26,27]
miR-15a-5p, miR-33b, miR-34a-5p, miR-9, miR-199a	Down	Downregulation of VEGF	[28]
miR-9, miR-34	Down	Inhibiting p53-suppressor protein-dependent proliferation in EC	[29]
miR-451	Down	Promoting pre-implantation embryogenesis by activating the Wnt signaling pathway	[30]
miR-196a	Up	Inhibiting the MEK/ERK signal and activating the progesterone receptor and decidualization in eutopic endometrium	[31]
miR-30c	Down	Regulating the expression of PAI-1 in eutopic and ectopic endometrium	[32]
miR-125b, miR-126-5p, miR-132-3p, miR-15b-5p, miR-152-3p, miR-155-5p, miR-181a-5p, miR-196b-5p, miR-199a-5p, miR-21-5p, miR-214-3p, miR-222a-3p, miR-23a-5p, miR-29b-3p, miR-98-5p	Down	Reducing inflammation in endometrial stromal cells (ESCs)	[33,34]
miR-138	Down	Reducing inflammation in endometriosis by minimizing levels of TNF-α, IL-1β, IL-6, and IL-18 through regulation of the NF-κB signaling pathway and VEGF	[35]
miR-20a	Up	Increasing production of prostaglandin PGE2 and enhancing the activity of extracellular signal-regulated kinases (ERK)	[36]
miR-200b, miR-200c	Down	Both miRNAs act as suppressive miRNAs on cancer cells. miR-200b has been shown to target TIMP-2 expression, and miR-200c targets FN1 (the fibronectin-coding gene) expression	[1]
miR-103	Up	Targeting the expression of TIMP-3 and stimulates tumor growth and invasion.	[1]
miR-199a, miR-122	Up	Positive correlation with IL-6 level	[28,36]
miR-29a-5p, miR-545-3p	Up	Stimulating angiogenesis by targeting the expression of VEGF	[1]
miR-199a-5p	Down	Reducing VEGFA expression, as well as decreased cell proliferation and angiogenesis	[28,37]
miR-424	Down	This molecule influences the activity of VEGFA, ERBB, mTOR, TGF-β, and PTEN/PI3K/AKT pathways, thereby affecting the process of tumor angiogenesis.	[38,39,40]
miR-15a-5p	Down	Downregulation of VEGF-A	[41,42]
miR-29b-3p	Down	Inactivating the MAPK/ERK and PI3K/AKT pathways.	[39,40]
miR-21-5p	Up	Promoting EMT in endometrial cancer cell lines	[43]
miRNA-20a	Up	Inducing angiogenesis by inhibition of the expression of DUSP2 and the induction of hyperexpression of genes related to ERK	[33]
miR-29c	Up	Desensitizing the cells of the endometrium to the impact of progesterone	[44,45]
miR-143-3p	Up	Desensitizing the cells of the endometrium to the impact of progesterone	[46]
miR-17	Up	Promoting cyclin expression through targeted downregulation of the gene phosphatase and tensin homolog (PTEN)	[47]
miR-23b, miR-99a, and miR-145	Up	Associated with embryo implantation defects	[48,49,50]
miR-323-3p	Up	Associated with ectopic pregnancy	[51,52]

**Table 3:** Newcastle–Ottawa Quality Assessment Scale and total score for each study, including case–control and cohort studies.

Studies	Newcastle–Ottawa Quality Assessment Scale Score								Score
	1	2	3	4	5	6	7	8	Total Number of Stars
**Case–Control Studies**									
[20]	A*	A*	A*	A*	A*	A*	B	A*	7
[21]	A*	A*	A*	A*	A*	A*	A*	B	7
[23]	A*	A*	A*	A*	A*	A*	A*	A*	8
[24]	A*	A*	A*	A*	A*	A*	A*	B	7
[25]	A*	A*	A*	A*	A*	A*	A*	B	7
[26]	A*	A*	A*	A*	A*	A*	A*	B	7
[29]	A*	A*	A*	A*	A*	A*	A*	B	7
[30]	A*	A*	A*	A*	A*	A*	A*	A*	8
[31]	A*	A*	A*	A*	A*	A*	A*	A*	8
[32]	A*	A*	A*	A*	A*	A*	A*	A*	8
[43]	A*	A*	A*	B	A*	A*	A*	B	6
[44]	A*	A*	A*	A*	A*	A*	A*	B	7
[45]	A*	A*	A*	A*	A*	A*	A*	B	7
[47]	A*	A*	A*	A*	A*	A*	A*	B	7
[48]	A*	A*	A*	A*	A*	A*	A*	A*	8
[50]	A*	A*	A*	A*	A*	A*	A*	A*	8
[49]	A*	A*	A*	A*	A*	A*	A*	A*	8
[52]	A*	A*	A*	A*	A*	A*	A*	B	8
**Cohort Studies**									
[51]	A*	A*	A*	A*	A*	A*	A*	B	8
[22]	A*	A*	A*	A*	B	A*	A*	A*	7

A*, one star; B, no star.

## Data Availability

The authors confirm that the data supporting the findings of this study are available within the article.
